# Role of Multidetector Computed Tomography Urography in Evaluating Obstructive Uropathy in a Tertiary Hospital in Rural India

**DOI:** 10.7759/cureus.70596

**Published:** 2024-10-01

**Authors:** Shreya Khandelwal, Rajasbala Dhande, Pratapsingh Parihar, Gaurav V Mishra, Anshul Sood

**Affiliations:** 1 Radiodiagnosis, Jawaharlal Nehru Medical College, Datta Meghe Institute of Higher Education and Research (DMIHER), Wardha, IND

**Keywords:** ct, hydronephrosis, obstruction, obstructive uropathy, radiology

## Abstract

Introduction: This study aims to evaluate the role of multidetector computed tomography (MDCT) urography in cases of obstructive uropathy to determine the cause, side, site, and level of obstruction and to differentiate between acute and chronic cases of obstructive uropathy based on imaging features.

Methods: Using Cochran's formula, a sample size of 121 patients was calculated. The patients underwent computed tomography (CT) urography to assess the obstructing agents causing obstructive uropathy. The conducted scan had four phases: the non-contrast phase, corticomedullary phase, nephrographic phase, and excretory phase. We assessed the obstructive agents and the changes they caused in the urinary tract.

Results: A total of 74 patients (61.16%) had calculus as their obstructive agent, followed by stricture (14.88%). The obstructive agents were intraluminal in 102 patients (84.3%) and extraluminal in 19 patients (15.7%). The ureter was the most common site of obstruction, accounting for 41.32%. The acute cases were 66 (54.55%), and the chronic cases were 55 (45.45%). A statistically significant (p<0.05) association was found using the chi-square test in the comparison of the enhancement and excretion of the kidneys and the type of case (acute or chronic). A statistically significant (p<0.05) association was found using the chi-square test in the comparison of the distribution of the secondary findings, such as perinephric fat stranding and perinephric fluid collection, and the type of case (acute or chronic).

Conclusion: MDCT urography is a highly reliable method of imaging the cause of obstructing agents in cases of obstructive uropathy and the damage caused by them. The type of enhancement and excretion and the secondary findings play an important role in determining the acuteness or the chronicity of the obstructive agent.

## Introduction

Obstructive uropathy is defined as the blockage in the flow of urine and may be either due to functional or anatomical abnormalities. The obstruction can occur at any site throughout the urinary tract involving the kidneys, ureter, urinary bladder, or urethra and is irrespective of age and can be categorized based on the site, side, duration, degree, and cause of obstruction. There are various etiological factors causing obstruction, including calculus, stricture, neoplasia, lymph nodal mass, ureterocele, and fibroid. Obstructive uropathy might cause abnormal dilatation of the ureter proximal to the obstructing agent and renal parenchyma [1-3]. Other possible outcomes of obstructive uropathy include a decrease in glomerular filtration rate, cortical cysts, glomerular hyalinization, and interstitial inflammation inside the kidney [4].

The obstructive agent can be assessed using various imaging modalities, including ultrasonography, X-rays, computed tomography (CT), and magnetic resonance imaging [5]. Ultrasound is highly effective in diagnosing the hydronephrosis caused by obstruction but is not highly efficient in detecting the cause of it because of imaging difficulties such as artifacts produced by the bowel gas and poorly opacified urinary tract due to decreased renal functioning [6]. Intravenous urography has been the gold standard imaging modality for the cases of obstructive uropathy for a long time. In the past decade, CT has been widely used and accepted due to its ability to detect nephrolithiasis and its ability to 3D reconstruct the whole scan [7,8].

This study aims to assess the role of multidetector computed tomography (MDCT) urography for the assessment of the obstructing agent causing obstructive uropathy and the changes in the urinary tract caused by it.

## Materials and methods

Study design and patients

This prospective, cross-sectional study was approved by our institutional ethics committee (approval number: DMIHER(DU)/IEC/2023/544). The sample size was calculated using Cochran's formula with an estimated proportion of 62%, an estimation error of 9%, and a confidence interval of 95%. A total of 121 patients aged between 10 and 85 years with imaging diagnoses of obstructive uropathy on CT urography were included in the study after obtaining detailed written and verbal informed consent. The patients were enrolled between July 2022 and May 2024. The patients with altered kidney function tests (serum creatinine greater than 1.4 mg/dL), patients with a prior history of contrast reactions, pregnant patients with simultaneous presence of obstructive uropathy, and patients not willing to give informed consent were excluded from the study.

Our primary aim was the efficacy of the CT urography in determining the cause, side, site, and level of obstruction. Our secondary aim was to differentiate the acute and chronic cases of obstructive uropathy based on the CT features.

Data collection and CT urography

The following data related to the patient were collected from the patient's medical and laboratory records: age, sex, clinical symptoms, duration of clinical symptoms, occupational and drug history, and serum creatinine.

All the patients had undergone four main phases of imaging: a non-contrast phase, a corticomedullary phase at 25-70 seconds of intravenous (IV) iodinated contrast administration, a nephrographic phase at 80-120 seconds post-IV contrast administration, and an excretory phase at 10-15 minutes post-IV contrast administration. The obtained data were reconstructed at 1.5 mm of slice thickness and interval.

The CT scan provided data on the cause, side, site, level, degree, size, and density of the obstructing etiology, the presence or absence of perinephric fat stranding, perinephric fluid collection, and organomegaly, and the pattern of enhancement in all the phases of CT urography.

Acute vs. chronic obstructive uropathy

Clinically, the patient will have a more severe and acute onset of symptoms in the acute setting of obstructive uropathy when compared with chronic obstructive uropathy.

The imaging features of acute obstructive uropathy include the presence of marked dilatation of the renal pelvis and calyceal system due to abrupt and severe obstruction. The parenchymal enhancement appears normal, reflecting the normal retention of blood supply by the renal tissues, suggesting a recent onset of obstruction. The secondary findings suggesting a drastic onset of obstruction include signs of inflammation and rapid response to the obstruction in the form of perinephric fat stranding and perinephric fluid collection. There are no significant parenchymal changes such as renal atrophy or the loss of renal functions in the acute onset obstructive uropathy [9].

In chronic obstructive uropathy, renal dilatation in the pelvis and calyceal system is mostly present and can be associated with a calyceal blunting appearance. The dilatation is more gradual and less severe when compared with that of acute obstruction. The renal parenchymal changes in the form of renal atrophy or scarring are mostly seen and are likely due to prolonged pressure on the renal tissue, which may cause loss of functional nephrons over time. The renal cortex may appear thinned out due to chronic compression and atrophy. Long-standing obstruction and reduced blood flow to the renal tissues may cause delayed or absent enhancement [9,10].

Statistical analysis

IBM SPSS Statistics for Windows, Version 22 (Released 2013; IBM Corp., Armonk, New York) was used to analyze the data obtained. Corticomedullary phase, nephrographic phase, excretory phase, etiology, site, and degree of obstruction were considered as the primary outcome variables. Perinephric fat stranding, perinephric fluid collection, and renomegaly were considered as the secondary outcome variables. Acute/chronic was considered as the primary explanatory variable.

Other relevant characteristics were age, gender, side, and presenting complaints. For quantitative data, mean and standard deviation were used for descriptive analysis; frequency and proportion were used for categorical variables. Quantitative variables that were not regularly distributed were characterized by median and interquartile range (IQR). Additionally, the Shapiro-Wilk test was used to evaluate the normal distribution. A p-value of >0.05 for the Shapiro-Wilk test indicated a normal distribution. The chi-square test and Fisher's exact test were used to evaluate categorical outcomes between study groups (Fisher's exact test was employed if the anticipated number in any cell was less than five or the sample size was less than 20). A p-value of less than 0.05 was deemed statistically significant.

## Results

The categorical outcomes between the study groups were evaluated between the study groups using chi-square and Fisher's exact test. Out of 121 enrolled patients, the mean age was 46.4 ± 14.3 years (between the ages 10 and 85), out of which 67.7% of patients were in the age group of 31-60 years. The total number of males was 74 (61.16%), and females were 47 (38.84%). The majority of patients came with complaints of either flank pain, difficulty in micturating, or pain in the abdomen (87.6%). Other less common complaints were hematuria, burning micturition, suprapubic pain, bilateral pain, weight loss, and dribbling of urine (Table 1).

**Table 1:** Descriptive analysis of presenting complaints in the study population (N=121).

Presenting Complaints	Frequency (%)
Pain in the right flank	40 (33.06%)
Difficulty in passing urine	24 (19.83%)
Pain in the left flank	22 (18.18%)
Pain in abdomen	20 (16.52%)
Hematuria	6 (4.95%)
Burning micturition	5 (4.13%)
Suprapubic pain	4 (3.31%)
Bilateral flank pain	3 (2.48%)
Difficulty in passing urine, bleeding per vagina	2 (1.65%)
Weight loss	2 (1.65%)
Dribbling of urine	1 (0.83%)
Fever	1 (0.83%)

Calculus was the most common obstructing agent present in 74 subjects (61.16%), followed by stricture in 18 patients (14.88%) and other less common etiologies, including bladder mass, lymph node mass, benign prostatic hyperplasia, ureterocele, bladder outlet obstruction, carcinoma cervix, duodenal mass, fibroid, leiomyosarcoma, psoas abscess, recto-vesical mass, retrocaval ureter, urethritis, and cystitis, as shown in Table 2. A total of 102 patients had obstructive uropathy due to intraluminal causes (84.3%) and 19 patients due to extraluminal causes (15.7%).

**Table 2:** Descriptive analysis of etiology/cause in the study population (N=121).

Etiology/Cause	Frequency (%)	
Calculus	74 (61.16%)	
Stricture	18 (14.88%)	
Bladder mass	5 (4.13%)	
Lymph nodal mass	4 (3.31%)	
Benign prostatic hyperplasia	3 (2.48%)	
Renal mass	3 (2.48%)	
Ureterocele	2 (1.65%)	
Bladder outlet obstruction	1 (0.83%)	
Carcinoma cervix	2 (1.65%)	
Duodenum mass	1 (0.83%)	
Fibroid	1 (0.83%)	
Leiomyosarcoma	1 (0.83%)	
Psoas abscess	1 (0.83%)	
Recto-vesical mass	1 (0.83%)	
Retrocaval ureter	1 (0.83%)	
Retroperitoneal mass	1 (0.83%)	
Ureter mass	1 (0.83%)	
Urethritis and cystitis	1 (0.83%)	

**Figure 1:**
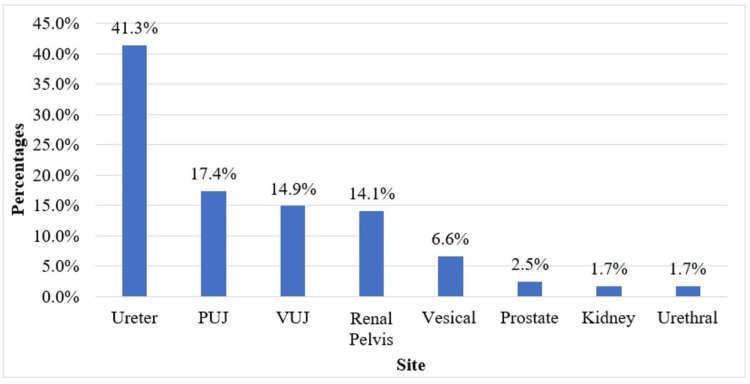
Bar chart of the site in the study population (N=121). N: number of subjects; PUJ: pelviureteric junction; VUJ: vesicoureteric junction

In all the subjects with obstructive uropathy, the ureter was the most commonly affected site, comprising 50 patients (41.32%), followed by 21 at the pelviureteric junction (17.36%), 18 at the vesicoureteric junction (14.88%), 17 at the renal pelvis (14.05%), eight at the urinary bladder (6.61%), three at the prostate (2.48%), two at the kidney (1.65%), and two at the urethra (1.65%), as shown in Table 3 and Figure 1. Out of 74 patients with calculus, 32 patients had calculus in the ureter (43.24%), 14 in the vesicoureteric junction (18.92%), 14 in the renal pelvis (18.92%), 11 in the pelviureteric junction (14.86%), two in the urethra (2.7%), and one in the urinary bladder (1.35%).

**Table 3:** Descriptive analysis of site in the study population (N=121). PUJ: pelviureteric junction; VUJ: vesicoureteric junction

Site	Frequency (%)
Ureter	50 (41.32%)
PUJ	21 (17.36%)
VUJ	18 (14.88%)
Renal pelvis	17 (14.05%)
Vesical	8 (6.61%)
Prostate	3 (2.48%)
Kidney	2 (1.65%)
Urethral	2 (1.65%)

**Figure 2:**
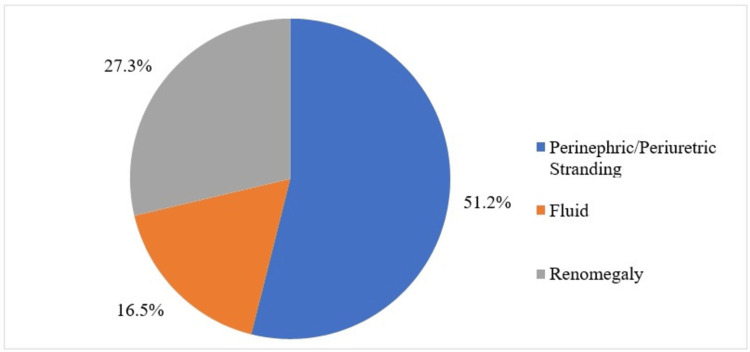
Pie chart of the distribution of the secondary findings in the study population (N=121). N: number of subjects

A total of 66 patients (54.55%) were categorized into acute obstructive uropathy and 55 patients (45.45%) into chronic obstructive uropathy based on the imaging features. Perinephric fat stranding was present in 62 patients (51.24%) and absent in 59 patients (48.76%). Perinephric fluid was present in 20 patients (16.53%) and absent in 101 patients (83.47%). Renomegaly was present in 33 patients (27.27%) and absent in 88 patients (72.73%), as shown in Table 4 and Figure 2.

**Table 4:** Descriptive analysis of the distribution of secondary findings in the study population (N=121).

Distribution of Secondary Findings	Frequency (%)
Perinephric fat stranding	-
Present	62 (51.24%)
Absent	59 (48.76%)
Perinephric fluid	-
Present	20 (16.53%)
Absent	101 (83.47%)
Renomegaly	-
Present	33 (27.27%)
Absent	88 (72.73%)

The corticomedullary and nephrographic phase showed hypo-enhancement in 33 patients (27.27%) and normal enhancement in 88 patients (72.73%). The excretory phase showed delayed excretion of contrast in 49 patients (40.50%) and normal excretion in 72 patients (59.50%), as shown in Table 5.

**Table 5:** Descriptive analysis of the different phases of computed tomography urography in the study population (N=121).

Corticomedullary Phase	Frequency (%)
Hypo-enhancement	33 (27.27%)
Normal enhancement	88 (72.73%)
Nephrographic phase	-
Hypo-enhancement	33 (27.27%)
Normal enhancement	88 (72.73%)
Excretory phase	-
Delayed	49 (40.50%)
Normal	72 (59.50%)

A statistically significant correlation (p<0.001) was found in the comparison of the distribution of perinephric fat stranding and acute/chronic obstructive uropathy. Comparison of the distribution of perinephric fluid collection and acute/chronic obstructive uropathy was statistically significant (p<0.05). However, no statistically significant correlation (p>0.05) was found in the comparison of the distribution of renomegaly and acute/chronic obstructive uropathy, as shown in Table 6.

**Table 6:** Comparison of the distribution of the secondary findings between acute and chronic (N=121). A p-value of <0.05 is considered statistically significant

Distribution of Secondary Findings	Acute/Chronic		Chi-square	p-value
	Acute (N=66)	Chronic (N=55)		
Perinephric fat stranding				
Present	48 (72.73%)	14 (25.45%)	26.833	<0.001
Absent	18 (27.27%)	41 (74.55%)		
Perinephric fluid				
Present	16 (24.24%)	4 (7.27%)	6.262	0.012
Absent	50 (75.76%)	51 (92.73%)		
Renomegaly				
Present	21 (31.82%)	12 (21.82%)	1.513	0.219
Absent	45 (68.18%)	43 (78.18%)		

**Figure 3:**
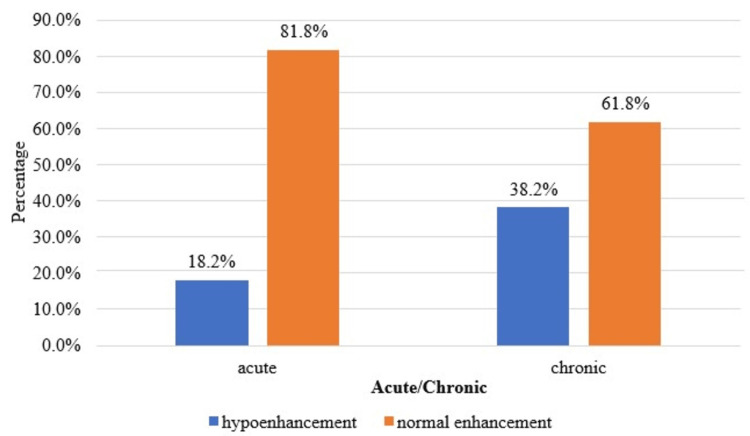
Cluster bar chart of the comparison of the corticomedullary phase between acute and chronic (N=121). N: number of subjects

**Figure 4:**
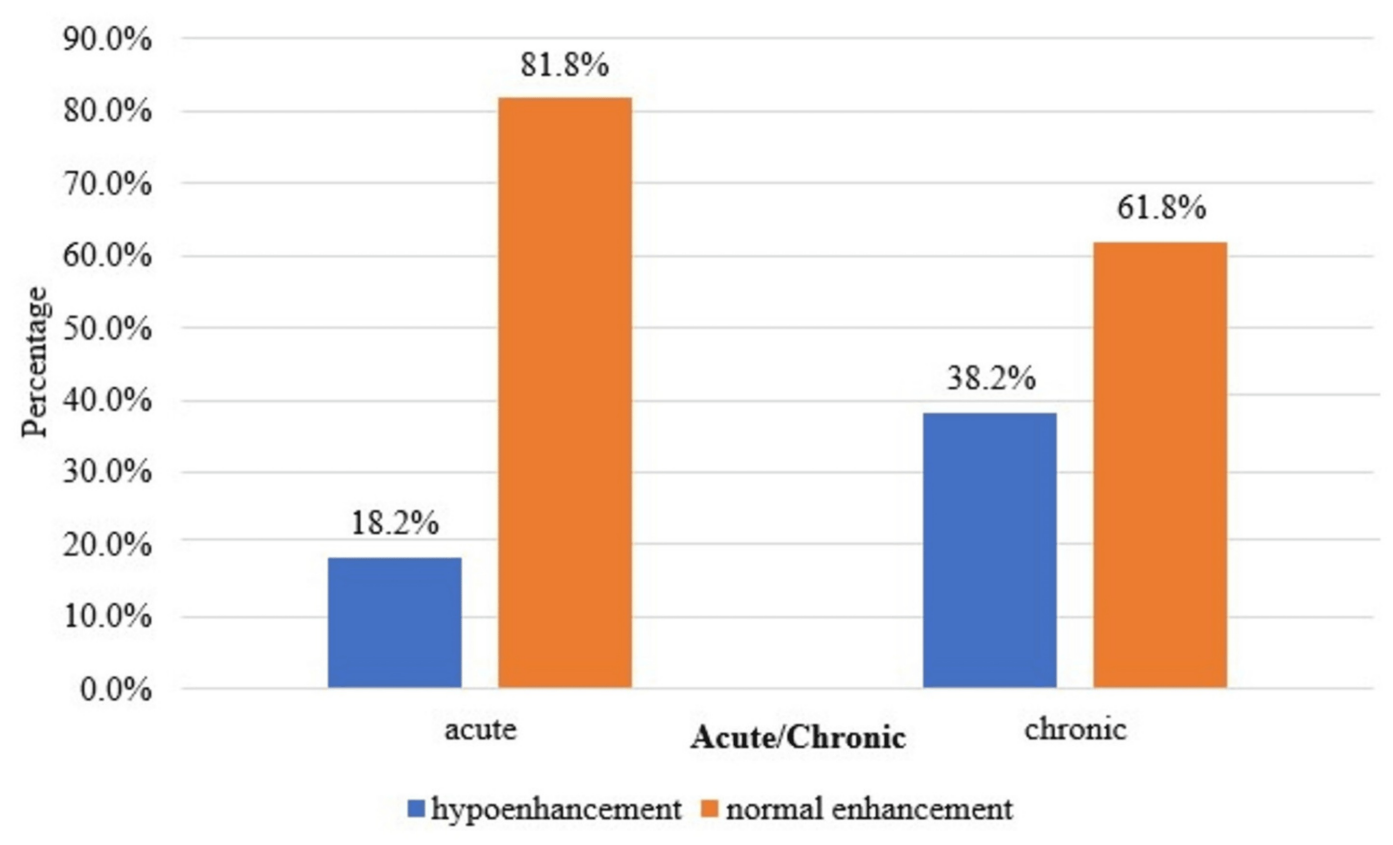
Cluster bar chart of the comparison of the nephrographic phase between acute and chronic (N=121). N: number of subjects

**Figure 5:**
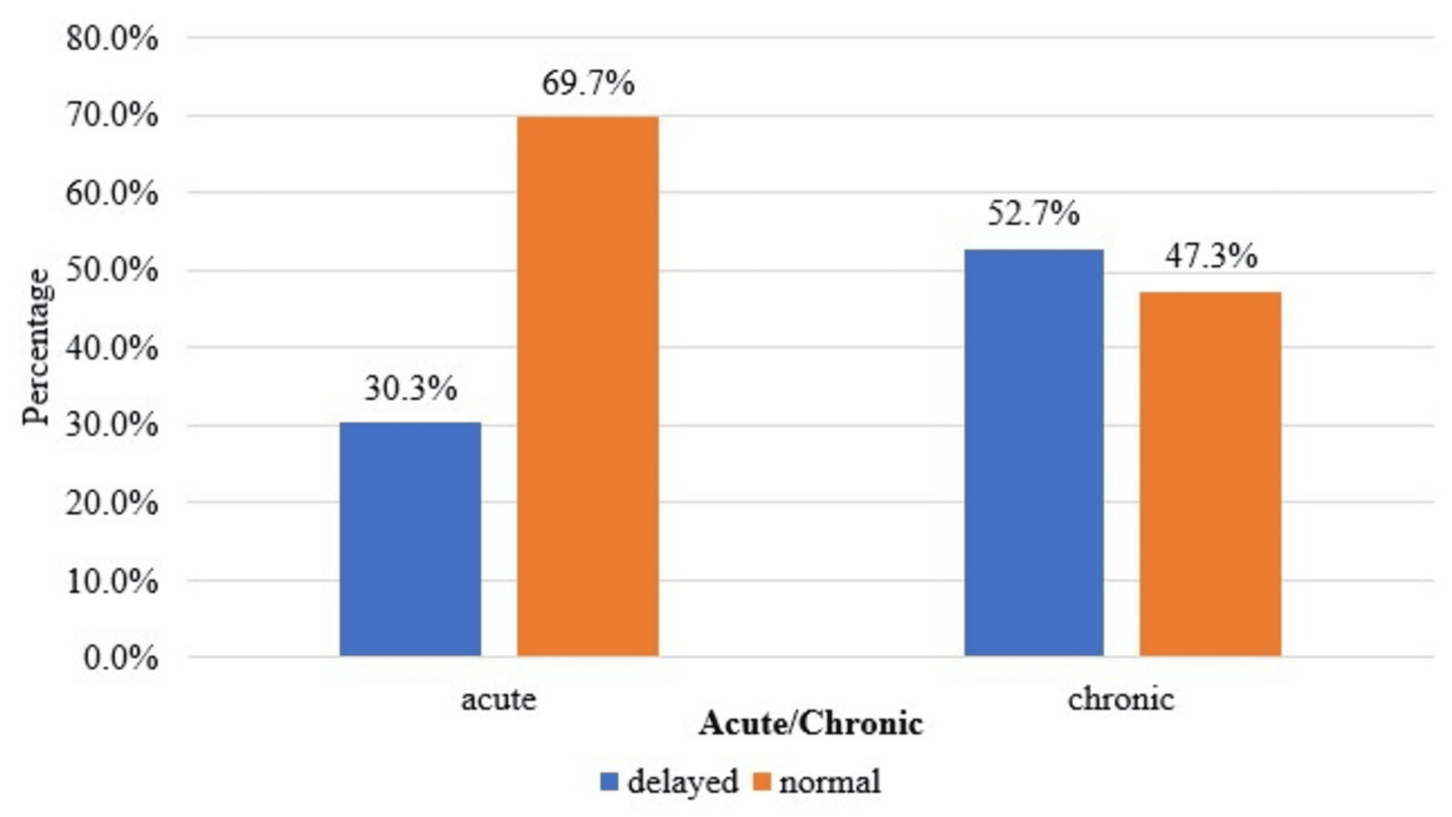
Cluster bar chart of the comparison of the excretory phase between acute and chronic (N=121). N: number of subjects

A statistically significant correlation (p<0.05) was found in the comparison of enhancement in the corticomedullary, nephrographic, and excretory phases and acute/chronic obstructive uropathy, as shown in Table 7 and Figures 3-5.

**Table 7:** Comparison of the different phases of computed tomography urography between acute and chronic cases (N=121). A p-value of <0.05 is considered statistically significant

Corticomedullary Phase	Acute/Chronic		Chi-square	p-value
	Acute (66)	Chronic (55)		
Hypo-enhancement	12 (18.18%)	21 (38.18%)	6.05	0.014
Normal enhancement	54 (81.82%)	34 (61.82%)		
Nephrographic phase	-	-	-	-
Hypo-enhancement	12 (18.18%)	21 (38.18%)	6.05	0.014
Normal enhancement	54 (81.82%)	34 (61.82%)		
Excretory	-	-	-	-
Delayed	20 (30.3%)	29 (52.73%)	6.26	0.012
Normal	46 (69.7%)	26 (47.27%)		

## Discussion

The present study was conducted involving 121 patients with obstructive uropathy. The mean age of the patients was 46.4 ± 14.3 years (between the ages 10 and 85 years), out of which 67.7% of patients were in the age group of 31-60 years. Kumar et al. [11] conducted a study involving 50 patients between the ages of 19 and 74 years, with 30% of the patients between 51 and 60 years. A study by Sharma et al. [12] on obstructive uropathy involved 50 patients ranging from 10 to 61 years with a mean age of 33.5 ± 14.3 years. These findings indicated a higher prevalence of obstructive uropathy in subjects of middle to older age groups.

The majority of the patients in our study were male (61.16%). In a study by Meenakumari et al. [13] and Sharma et al. [12], 59.6% and 62% of the patients were male. This high prevalence of obstructive uropathy could be due to anatomical differences such as prostate gland, length, and structure of the urethra.

Calculus was the main etiological factor (61.16%) in our study, followed by strictures (14.88%). Similar to the study by Youssef et al. [14] involving 50 patients and 65 ureters, it was found that 41.5% of the patients had calculus as an etiological factor causing obstructive uropathy. In the present study, 102 patients (84.3%) had intraluminal causes of obstruction, and 19 patients (15.7%) had extraluminal causes of obstruction, indicating that the causative agent of obstructive uropathy lies predominantly within the urinary tract. Within the urinary tract, the ureter (41.32%) was the most commonly affected site, among which the obstruction in the proximal ureter was seen in 44%, the middle ureter in 38%, and the distal ureter in 18% of the patients. Similarly, a study by Youssef et al. [14] reported that 58.46% of patients had obstructive agents in the distal one-third of the ureter.

The corticomedullary phase is useful for identifying cortical abnormalities, the nephrographic phase is important for identifying parenchymal lesions, and the excretory phase is important for diagnosing blockages in the urinary tract.

In the present study, there was a statistically significant (p<0.05) association between the type of case (acute or chronic) and the pattern of enhancement in the corticomedullary and nephrographic phases and the pattern of contrast excretion in the excretory phase. Sharma et al. [12] observed a decreased enhancement in the nephrographic phase in patients with substantial parenchymal involvement and chronic renal disease. There is a uniform loss of enhancement in this phase, suggesting a diffuse disease process. Moawad et al. [15] conducted a study on 30 patients undergoing CT urography to evaluate obstructive uropathy and found that delayed excretion of contrast was often associated with ureteral stones and tumors.

A statistically significant correlation (p<0.05) was found between the presence of perinephric fat and perinephric fluid collection and the type of case (acute or chronic). However, there was no statistically significant correlation between the acute or chronic cases and renomegaly. We did not find any recent studies that have shown this correlation. From the outcome of this correlation, it was understood that the perinephric fat stranding and perinephric fluid collection were mostly observed in cases of acute obstructive uropathy. It can act as an important marker for understanding the disease progression and approximate duration in cases with inadequate medical history. This finding could help improve the prognosis by adequately assessing and starting the management in a timely manner.

The limitations of the study include its two-year period, which may not adequately capture the long-term trends and variances in the etiology of obstructive uropathy, necessitating a longer study duration for comprehensive analysis. Additionally, the study was conducted in a single hospital, thus reflecting the findings of a particular geographic area and healthcare environment, warranting a multi-centric study.

## Conclusions

This study was conducted to emphasize the role of MDCT urography in diagnosing obstructive uropathy. In the present study, it was observed that calculus was the most common etiological factor causing obstructive uropathy. The ureter was the most commonly affected site. A statistically significant (p<0.05) association was found between the type of case (acute or chronic) and the pattern of enhancement and excretion in the corticomedullary, nephrographic, and excretory phases. These findings underscore the critical role of CT urography over non-contrast CT scans in diagnosing and managing obstructive uropathy. Furthermore, our study observed a statistically significant (p<0.05) association between the type of case (acute or chronic) and the presence of perinephric fat and perinephric fluid collection, which has not been demonstrated in the recent literature and may aid in the diagnosis of acute and chronic obstructive uropathy.
